# Promoting sleep health during pregnancy for enhancing women’s health: a longitudinal randomized controlled trial combining biological, physiological and psychological measures, Maternal Outcome after THERapy for Sleep (MOTHERS)

**DOI:** 10.1186/s40359-024-01827-1

**Published:** 2024-06-10

**Authors:** Debora Meneo, Elisabetta Baldi, Silvia Cerolini, Sara Curati, Stefano Bastianini, Chiara Berteotti, Giuliana Simonazzi, Mauro Manconi, Giovanna Zoccoli, Paola De Bartolo, Francesca Gelfo, Viviana Lo Martire, Chiara Baglioni

**Affiliations:** 1 Department of Human Sciences, Guglielmo Marconi University, Rome, Italy; 2 Department of Biomedical and Neuromotor Sciences, University of Bologna, Bologna, Italy; 3 Department of Medical and Surgical Sciences, University of Bologna, Bologna, Italy; 4 Sleep Medicine Unit, Faculty of Biomedical Sciences, Neurocenter of the Southern Switzerland, Regional Hospital of Lugano, Università Della Svizzera Italiana, Lugano, Switzerland; 5 Dot. Of Neurology, Bern University, Bern, Switzerland; 6 IRCCS Fondazione Santa Lucia, Rome, Italy; 7 Department of Clinical Psychology and Psychophysiology, Centre for Mental Health (Department), Medical Center, Faculty of Medicine, University of Freiburg, Freiburg, Germany

**Keywords:** Perinatal sleep quality, Insomnia, Pregnancy, Post-partum, Emotional processes, Actigraphy, Sleep diary, Cortisol, Stress

## Abstract

**Background:**

Sleep is vital for maintaining individuals’ physical and mental health and is particularly challenged during pregnancy. More than 70% of women during the gestational period report insomnia symptoms. Sleep dysfunction in the peripartum increases the risk for a cascade of negative health outcomes during late pregnancy, birth, and postpartum. While psychological interventions are considered the first line treatment for sleep difficulties, they are still scarcely offered during pregnancy and there is a lack of longitudinal research combining psychological and physiological indices.

**Methods:**

The present protocol outlines a randomized controlled trial aimed at testing the long-term effectiveness of an automatized digitalized psychoeducational intervention for insomnia for expectant mothers complaining insomnia symptoms without comorbidity. Outcomes include physiological, hormonal, and subjective indices of maternal psychopathology, stress, and emotional processes, and sleep and wellbeing of the family system.

The trial is part of a longitudinal study evaluating expectant mothers from early pregnancy (within the 15th gestational week) to 6-months postpartum through 6 observational phases: baseline (BSL), 6- and 12-weeks from BSL (FU1-FU2), 2-to-4 weeks after delivery (FU3), and 3- and 6-months after delivery (FU4-5). We plan to recruit 38 women without sleep difficulties (Group A) and 76 women with sleep difficulties (Group B). Group B will be randomly assigned to digital psychological control intervention (B1) or experimental psychoeducational intervention targeting insomnia (B2). At 3 time points, an ecological-momentary-assessment (EMA) design will be used to collect data on sleep and emotions (diaries), sleep-wake parameters (actigraphy) and stress reactivity (salivary cortisol). We will also test the DNA methylation of genes involved in the stress response as biomarkers of prenatal poor sleep. Information on partner’s insomnia symptoms and new-borns’ sleep will be collected at each stage.

**Discussion:**

The proposed protocol aims at testing an easily accessible evidence-based psychoeducational intervention for expectant mothers to help them improving sleep, health, and wellbeing in the peripartum. The results could improve the understanding and management of sleep difficulties and peripartum depression.

**Trial registration:**

The study protocol has been registered on 22 April 2024 with ClinicalTrials.gov Protocol Registration and Results System (PRS), ID: NCT06379074. Protocol version: April 23, 2024.

**Supplementary Information:**

The online version contains supplementary material available at 10.1186/s40359-024-01827-1.

## Background

Improving maternal mental health is a worldwide health priority [1]. Nevertheless, several scientific sources highlighted the lack of empirical data which could drive clinical practice. Mental disorders are generally more prevalent in women than in men, especially considering specific sensible life periods, as peripartum [2, 3]. Nevertheless, pregnant and breastfeeding mothers are often excluded from biomedical research and almost neglected from clinical trials for safety reasons [4]. This partially explains lack of evidence-based research leading to effective preventive care for expectant mothers [4]. Peripartum psychopathology is highly prevalent and it is associated for all family’s members with negative health outcomes [5, 6]. Emotional and psychological peripartum wellbeing is protective for post-partum mental health, emotion regulation and maternal-fetal attachment, influencing not only mother-child attachment but also child’s later development and the emotional balance of the whole family [7]. Symptoms of insomnia, that peaks just in the immediate postpartum period, are linked with increased risk for psychopathology and somatic disorders [8–12]. Around 70% of women reported a low quality of sleep in the first month after delivery [12]. The National Sleep Foundation reported that up to 84% of women complain symptoms of insomnia during peripartum [13]. Insomnia during pregnancy is interpreted as a result of physical, endocrinological, and emotional changes and as risk factor for increased stress and psychophysiological negative outcomes, including difficulties with mother-infant attachment relationship [14]. A meta-analysis [15], based on 9 studies, reported significant correlations between perinatal insomnia and depressive symptoms. Despite high prevalence and association with several negative outcomes, there is lack of longitudinal studies which assess the relationship between insomnia during pregnancy, peripartum stress and psychopathology combining physiological and subjective outcomes [16, 17]. Furthermore, clinically, insomnia complaints during pregnancy remain underdiagnosed and undertreated [18]. The persistence of sleep problems during pregnancy represents a stressing condition which might have long-term consequences on child’s behaviour [19]. Indeed, stressing factors during peripartum lead to permanent changes in the fetus that might predispose the individual to disease later in life. Several studies showed the negative consequences of perinatal stress on neurogenesis, metabolism and the cardiovascular system in adult offspring [20], while only few data indicate that perinatal stress may also have consequences for adult sleep and cognitive phenotypes [21]. Maternal stress produces overexposure of the fetus to glucocorticoids which epigenetically impairs the development of the hippocampus, dampening its inhibitory role in the hormonal stress response. The exacerbation of the stress response in adult offspring leads to high levels of glucocorticoids, which are known to exert central effects on sleep and cognition [21]. Hypercortisolemia also impacts on FKBP5 (a gene encoding a glucocorticoid receptor co-chaperone) methylation in the blood and the hippocampus of human and mouse model of post-traumatic stress disorder [22–24]. Thus, maternal insomnia may contribute to triggering this cascade of events and have long-term consequences for health, including consequence on adult sleep phenotype.

Early psychological interventions are feasible and have been evidenced to fit with expectant mothers’ preference for health care, in a period of life in which psychotropic pharmacotherapy may be associated with patients and doctors’ concerns [25]. Cognitive-Behavioural Treatment for Insomnia (CBT-I) is the first-line treatment for insomnia and the most effective in the long term even when compared with the used hypnotics [26]. CBT-I targets those factors that maintain insomnia over time, such as dysregulation of sleep drive, sleep-related anxiety, and sleep-interfering behaviours [27]. Main CBT-I strategies include establishing a learned association between the bed and sleeping through stimulus control, restoring homeostatic regulation of sleep through sleep restriction, and reducing anxious sleep-related thoughts through cognitive restructuring. Preliminary evidence suggests that CBT-I is effective for curing insomnia during pregnancy and reduces depressive symptoms [28]. Although CBT-I is the first line treatment for insomnia disorder, there are barriers that limits its implementation, including the lack CBT-I providers and patients’ difficulties in accessing treatment [29]. Digital CBT-I (dCBT-I) is intended to meet the demand for CBT-I through the offering of treatment through digital technology [30]. dCBT-I reduces costs, increase scalability and has been proven to be effective [31, 32], becoming a recommended treatment for insomnia disorder [26]. Pregnant women may be met with additional barriers to CBT-I, including the need to menage prenatal health appointments and to reserve medical leave for them [33].

Though CBT-I has been proved to lead to long-lasting enhanced benefits compared to sleep pharmacotherapy in all adults [26], this difference may be even more relevant for pregnant women. Indeed, women and clinicians may be not prone to use medications during pregnancy due to possible negative outcomes [34]. All hypnotics available on the market and with an indication for insomnia belong to the category C (FDA, food and drug administration), for which risk cannot be ruled out because there are no satisfactory studies in pregnant women.

Psychological interventions guide patients in increasing stress regulatory skills, which may enhance resilience and efficacy in the whole peripartum period, leading to positive outcomes for the new parent-child relationship.

### Study aims

The present trial aims to evaluate the long-term effectiveness of digital psychoeducational module based on CBT-I for expectant mothers complaining insomnia symptoms without comorbid somatic or mental disorders on:

- physiological, biological, genetical and subjective indices of maternal psychopathology (perinatal depression and anxiety), stress, and emotional processes; these will be assessed through cortisol levels, recording of the sleep-wake activity through actigraphies, sleep diaries, and online questionnaires;
- father’s and new-born’s sleep and perceived parental stress; these will be assessed through online questionnaires and sleep diaries.

### Hypotheses

#### Primary hypotheses

At post-treatment, participants in the intervention group, compared to control group, will show improvement in sleep health (physiological and self-reported) and in indices of stress and psychopathology (biological and self-reported), including perinatal depression (PND) and anxiety. Improvement in biological and self-reported indices of psychopathology and stress will be mediated by improvement in insomnia symptoms and sleep dimensions.

#### Secondary hypothesis

The improvement will be maintained at follow-ups.

## Methods

### Study design

Randomized controlled trial with three parallel arms (intervention, active control group, passive control group) with allocation ration 1:1 and 5 follow-up assessments following pregnant women from early pregnancy (within the 15° gestational week) to 6-months after birth. Three of the six phases of the study (Baseline, follow-up1 and follow-up5) will also include Ecological Momentary Assessment (EMA).

The protocol follows the Standard Protocol Items: Recommendations for Interventional Trials (SPIRIT) statement (see SPIRIT checklist available as Supplementary File S1).

### Participants

Women will be mainly recruited in the middle Italy area of Bologna and Rome (Italy) and study’s materials and sensitive data will be crepitated and archived in the Department of Biomedical and Neuromotor Sciences, University of Bologna (Italy). Different recruitment procedures will be used. Firstly, informative flyers will be distributed to women contacting public services for care through collaboration with the Unit of Obstetrics and Gynaecology of the S. Orsola Hospital in Bologna. Women assessed for the first ultrasound check will be invited to participate. Secondly, informative flyers will be distributed to private gynaecologists, personal contacts of the research group, and pharmacies. The flyer will contain a summary of the study, the contacts of the researchers and a QR-code to a website created for the study (www.studiomothers.it). The website will contain further detailed information on the study rationale, aim and procedure. Interested women will be given the possibility to fill a contact form in the website; they will then be asked for their e-mail or telephone number to be contacted for the first appointment with the researcher for the screening procedure (see below in “Procedure”).

Women will be continuously recruited for circa 8 months, starting May 2024, until reaching the required sample size of 150 (see power analysis below).

Inclusion criteria will be:

- age ≥ 18 yrs. old;
- ≤ 15th week of pregnancy at the time of recruitment;
- good knowledge of Italian language;
- no severe diagnosis of relevant somatic or mental disorder (ascertained with clinical interview);
- no smoking;
- no alcohol intake;
- no assumption of illegal drugs;
- BMI ranging 18–30 (i.e., not presenting obesity or underweight following international criteria; WHO).

During the trial, participants will have no restriction to their usual care (e.g., scheduled prenatal health appointments). They will be allowed to receive concomitant care for other mental health problems (not insomnia) after enrolment.

### Sample size

To estimate required sample size for adequate power of the clinical trial, we used as primary outcome peripartum depression (PDS) at post-treatment. A study that compared scores on the Edinburgh Postnatal Depression Scale (EPDS) in 132 women divided into two groups, cognitive-behavioral therapy for insomnia (*N* = 89) or control group (*N* = 43), before, during and after pregnancy [35] was used. The software G-Power was used to estimate sample size: 114 women in total would be needed to have an effect power of at least 80%. Drop-out rates in previous studies were around 5 to 16% for post-treatment and short follow-ups [28, 33] and around 17% for 6 months follow-up [36]. We plan to recruit 150 participants, accounting for a dropout rate of 24%.

### Screening

Following our recruitment strategy, all women at the first ultrasound check or all women expressing interest through the study’s website will be contacted for an appointment with a clinical psychologist for the screening, which will be conducted in a confidential space in a room at the Universities’ Department involved (Department of Biomedical and Neuromotor Sciences, University of Bologna; Department of Human Sciences, Guglielmo Marconi University of Rome). This space will be used for all in-person contact with the participants (detailed below). Study’s materials, including biological samples, will all be conserved labelled with pseudonymized codes (number and letters without personal information) in a secured room in the Department of Biomedical and Neuromotor Sciences, University of Bologna for the duration of the study. A member of the research team will explain study aims and procedure. All women will be asked to read and sign the informed consent before proceeding. A separate informed consent will be asked for the use of salivary samples for the analyses of cortisol level and DNA-methylation (see “Baseline” section). Screening will be evaluated through a psychological structured interview (Structured Clinical Interview for DSM-5, SCID-5 in the brief version QuickSCID-5) [37] and a specific interview about their sleep [38] by clinical psychologists (D.M., E.B., S.Ce.). Information on pregnancy and socioeconomic variables will be collected. Women will be asked to share along with their consent, gynaecological medical data on their health status (e.g. information on pregnancy).

Eligible participants will be divided in two groups:

1. *Group A*: control group of healthy pregnant women with no insomnia complaints (*N* = 38);
2. *Group B*: pregnant women complaining of subthreshold or clinical insomnia (*N* = 76).

Insomnia categories (absence of insomnia; subthreshold insomnia; clinical insomnia) will be operationalized using the validated questionnaire ‘Insomnia Severity Index’ (ISI) [39]. ISI is a measure of insomnia severity in the 2 weeks before compilation. Participants provide answers on a five-point Likert scale on 7 items. The score is the simple sum of the answers to all the questions and can range from 0 to 28. The total score is interpreted as follows: no insomnia (0–7); subthreshold insomnia [8–14]; clinical insomnia [15–28].

Group A (passive control group) will serve as a comparator for the absence of any intervention, accounting for natural changes in sleep during pregnancy.

### Randomization

Participants in Group B will be randomly assigned to one of the following two subgroups:

*Subgroup B1* (active control): psychological placebo (*N* = 38);

*Subgroup B2* (intervention): psychoeducation based on CBT-I adapted to pregnancy (*N* = 38).

Allocation concealment will be performed in steps: firstly, a code has to be created for each future participants to set the online platform they will use. The alphanumeric code (here referred to as “ID-code”) is composed of a string of numbers and the subgroup (B1 or B2). The ID-codes will be created before the start of the study by a computerized generator of random numbers. Each participant, upon allocation, will receive a folder with their ID-code, together with the instruments and information for the baseline phase (see below). A member of the research team who will not conduct screening and assignment to groups will prepare the sealed folders containing the ID-codes. The folders of Group B will be shuffled and then sequentially numbered. After the screening, each participant entering the study in the Group B will be given a numbered folder labelled with the letter “B”. With this procedure, participants will be randomly assigned to subgroup B1 or B2, concealing allocation to both participants and the investigator. Neither the psychologists conducting the screening interviews, nor the participants will know the allocation until after the screening.

Participants in the passive control condition (*Group A*) will also receive a randomly generated ID-code.

Participants will be given the credentials to log-in to their personal area on the website of the study for the next phases. The credentials will match the ID-code and thus each condition will have access to the respective online contents. The ID-codes will be used for each subsequent phase of the study.

### Blinding

The screening procedure, including the clinical interviews, will be conducted before group allocation. Members of the research team scoring actigraphic data and analysing salivary samples will be blinded to participants’ allocation. Statistical analysis will be conducted on participants’ data associated with the random number labelled with the ID-code without the letter identifying the group allocation.

### Procedure

The study includes a screening phase for ascertainment of eligibility criteria and 6 phases (one baseline and 5 follow-ups).

**Fig. 1:**
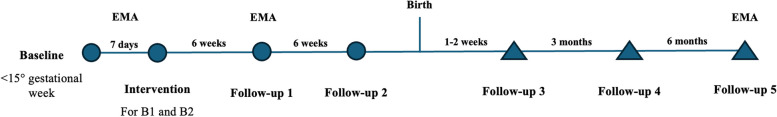
Study timeline. Legend: Circles indicate prepartum assessments; triangles indicate postpartum assessment. EMA = Ecological Momentary Assessment

All women will be observed longitudinally in 6 assessment evaluations (see Fig. 1):

1. Baseline: between 11th and 15th pregnant week;
2. Follow-up-1: after 6 weeks from baseline;
3. Follow-up-2: after 12 weeks from baseline;
4. Follow-up-3: 1-to-2-week after birth;
5. Follow-up-4: 3-months post-partum;
6. Follow-up-5: 6-monhts post-partum.

**Fig. 2:**
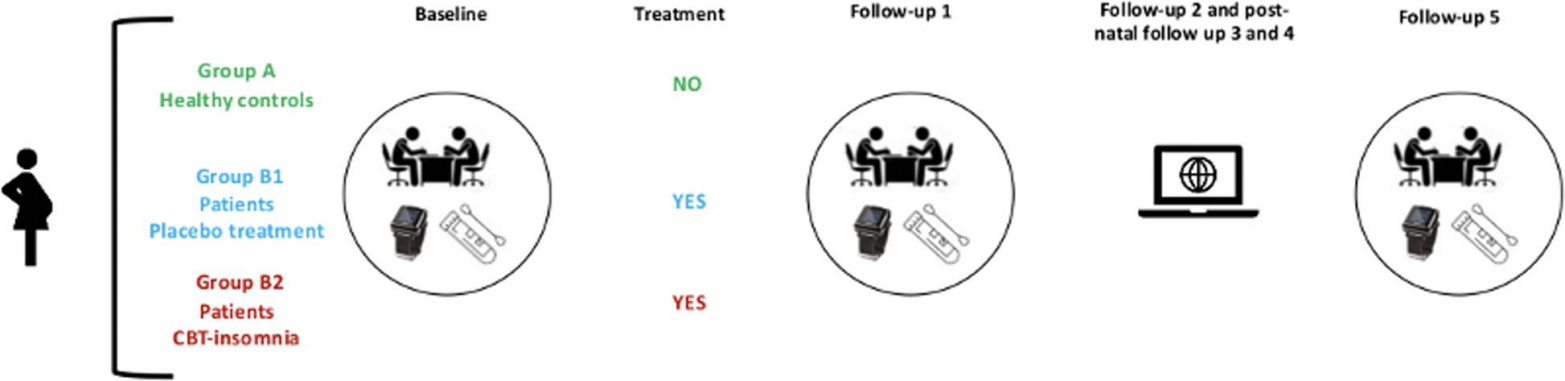
Study procedure

For women who will be offered clinical treatment (*Group B*), baseline and follow-up-1 assessments will be conducted pre- and post-treatment (right after the 6 weeks of intervention). Partner of each participant women will be also invited to take part in the study. Their participation will include filling out online questionnaires and sleep diaries for each assessment point detailed above. At each time point of the study, participants’ will be contacted via e-mail for a reminder to log-in to their personal area of the website and complete assigned instruments and/or schedule an appointment with a member of the research team to receive the material for the observational week (see “EMA week”). The procedure is synthetized in Fig. 2.

The full procedure of the trial will be monitored by the study coordinator (CB). Weekly appointments will be held between the coordinator and the primary investigators to review recruitment, randomization, data collection, and data analysis.

### Baseline

All participants will be asked to log-in to the study website using their personal credentials and to fill in a set of questionnaires (described in “Instruments”). Participants’ sleep and emotional processes will be monitored for 7-days through an ecological-momentary-assessment (EMA) design, which allows repeated experience sampling [40]. The EMA week will include: sleep and emotions diaries (in the morning and evening) to be completed on the website; an actigraphic (wearable wrist device) monitoring sleep-wake variables for 7 days. Under express and separate consents, on the first day of the EMA week, women will be trained to collect, at their home, saliva sample through the saliva swabs they received in their folder. Collection will be carried on in 2 time-points during the day: immediately after awakening and just before bedtime. The saliva sample will be used for evaluation of cortisol levels and for the analysis of DNA methylation of genes involved in the stress response: the FKBP5, the BDNF, and the NR3C1 [41]. Rationales for these analyses are described in “Outcomes”.

Participants will store their samples at home. At the end of the EMA week, participants will be given appointment with the researchers to bring the actigraphy and the saliva samples, closed in an envelope and already marked with unique ID-code.

Participants will be explained that their partners could also take part to the study under their expressed informed consent. Women will be given an informative form directed to partners. Within 3 days from the screening, participants will be contacted via e-mail to assess if their partners are interested to participate. In case of interest, partners’ contacts will be asked. Interested partners will receive an e-mail with their unique credentials to log-in to the website and express their informed consent. In their personal area of the website they will be asked to complete, at each phase of the study, a set of questionnaires: socio-demographic variables for Baseline; the ISI for Baseline and Follow-ups 1–5, a 7-day sleep diary for Baseline and Follow-ups 1 and 5; the Parenting Stress Index (PSI) [42] at Follow-ups 3–5. Participants and partners will be matched through an ID-code and will access their respective unique personal area on the website.

### Intervention

*Group A* (ISI score under 8) will be observed longitudinally through standard of care above-described procedure, without an active intervention. *Group B* (ISI score above 7) will be randomly assigned to 2 active interventions both conducted through digital therapy: (1) psychoeducation based on CBT-I adapted for pregnancy (experimental intervention; subgroup B2); (2) information on sleep disorders and on peripartum-related issues (placebo intervention; subgroup B1).

Each intervention will share the following characteristics:

- fully digitalized on the study website;
- composed of 6 weekly 20-minutes online sessions;
- each session will include a before and after brief survey to monitor satisfaction;
- each participant will start the intervention upon completion of the baseline assessment;
- participants will be asked to fill out directly on the website a reduced version of the sleep and emotion diaries each day during the intervention (6 weeks).

For Group B1 (placebo), each session will include: educational videos (ca. 20 min) on aspects related to pregnancy and sleep; brief feedback questions. Participants in the placebo intervention will not be given specific indications on skills or techniques for sleep difficulties and will not have access to the weekly chat with the clinician.

Sessions will cover the following contents:

- Session 1: phases of pregnancy;
- Session 2: sleep disorders;
- Session 3: nutrition and physical activity during pregnancy;
- Session 4: childbirth;
- Session 5: psychophysical development of the child in the first year of life;
- Session 6: synthesis of previous sessions.

For Group B2 (intervention), each session includes: brief videos delivering the intervention content (ca. 20–30 min); short questions aimed at raising awareness of how the content of the videos related to participants’ experience (e.g., one own dysfunctional beliefs and attitudes about sleep); short feedback questions; downloadable information (pdf) on sleep education and CBT-I derived techniques adapted to pregnancy. The downloadable information associated with each session are chapter of a psychoeducational book (the table of contents, which follows the video contents, are available in Supplementary file S2). Each session would require around 30 min a week. Participants will be given opportunity to raise concern or difficulties with the content of the intervention and with the filling of sleep diaries, both as feedback before and after each session and with weekly opportunity to directly chat with a clinician through the web site.

- Session 1: Presenting the aims of the intervention and introducing psychoeducation on physiological regulation of sleep, on sleep health and on how sleep changes during pregnancy;
- Session 2: Psychoeducation on psychological regulation of sleep; impact of behaviours on sleep regulation; introduction on sleep compression and stimulus control;
- Session 3: Psychoeducation on cognitive factors maintaining sleep difficulties; introduction on cognitive restructuring;
- Session 4: Psychoeducation on emotional factors maintaining sleep difficulties and on the bidirectional association between sleep and emotions; introduction “into” or “on” emotion regulation techniques;
- Session 5: Psychoeducation on sleep in the postpartum and on the development of sleep regulation in children;
- Session 6: Relapse prevention and focus on acquired skills and on how to prioritize sleep.

Adherence will be monitored through weekly chat with a licensed psychotherapist expert in CBT-I (C.B.) and through diary completion during the six weeks of the intervention. Strategies to improve adherence will be implemented during the weekly chat and include: reviewing week sleep diaries, identifying barriers and resistance, rediscussing any technical difficulties with the online sessions. The availability of direct contact will also serve to assess any difficulty with specific psychoeducational content and to modulate the intervention accordingly.

#### Follow-up-1

After 6 weeks from baseline (post-treatment for *Group B*), participants will be assessed trough the sleep and emotions diaries and the actigraphic monitoring. Participants who consented to the treatment of salivary samples will be asked to collect their sample through salivary swabs the first day of EMA in the 2 time-points of morning and evening. Participants will also be asked to fulfil a set of questionnaires (described in “Assessments”). The EMA week will thus follow the same procedure of the baseline assessment.

#### Follow-up-2

After 12 weeks from baseline, participants will be asked to log-in to their personal area on the website and complete a set of questionnaires.

#### Follow-up-3

Between 1 and 2-week after birth, participants will be asked to log-in to their personal area on the website and complete a set of questionnaires, including information on birth (type of birth, length of labour, self-perception of stress and fatigue during birth) and on the newborn (weight, Apgar Index, specific problems after birth). Information on feeding modality and mothers’ related feeling will be collected.

#### Follow-up-4

Three months post-partum, participants will be asked to log-in to their personal area on the website and complete a set of questionnaires. Specific information on the experience of motherhood will be asked, including: mother-infant attachment, maternal perceived stress, feeding modality and maternal related feeling. Paternal perceived stress will also be part of the assessment.

#### Follow-up-5

Six months post-partum, participants will be again observed trough the EMA week, following the procedure of the Baseline and Follow-up 1 phases, with also a sleep diary for their children. Participants will also be asked to fill a set of questionnaires, including information on the child’s temperament and on maternal and paternal experiences.

### Outcomes

#### Primary outcomes

- Stress reactivity: salivary cortisol level assessed by saliva sample provided by participants through swabs twice a day once at baseline, follow-up 1 and 5.
- Sleep efficiency: Total Sleep Time (min)/Time In Bed (min) expressed in percentage and assessed trough actigraphy monitoring. Assessed one week at baseline, follow-up 1, follow-up 5.
- Depressive symptoms: Edinburgh Postnatal Depression Scale (EPDS) [43] total score. EDPS scores > 10 will be considered as indicative of PND. Assessed at baseline, follow-up 1 and 5.
- Mothers’ Insomnia symptoms: Insomnia Severity Index (ISI) [39] total score. Assessed at baseline, follow-up 1–5.
- Anxiety symptoms: Generalized Anxiety Disorder questionnaire (GAD-7) [44] total score. Assessed at baseline, follow-up 1–5.
- Valence of affective states at morning and evening: visual scale in sleep and emotional diaries. Assessed one week at baseline, follow-up 1, follow-up 5.
- Arousal of affective states at morning and evening: visual scale in sleep and emotional diaries. Assessed one week at baseline, follow-up 1, follow-up 5.
- Emotion regulation strategies: Cognitive Emotion Regulation Questionnaire – Italian Short-Version (CERQ-IS) [45] subscales scores. Assessed one week at baseline, follow-up 1, follow-up 5.

#### Secondary outcomes

- Maternal post-partum stress: Parenting Stress Index (PSI) [42] total score. Assessed at follow-up 3–4.
- Fathers’ Insomnia symptoms: ISI total score. Assessed at baseline, follow-up 1–5.
- Children sleep difficulties: Brief Infant Sleep Questionnaire (BISQ) [46] total score. assessed at follow-up 3–5.

#### Additional outcomes

- Biomarkers of prenatal sleep alteration: DNA methylation of the genes FKBP5, BDNF and NR3C1 (see “Salivary samples”). Assessed at baseline.

### Assessments

#### Self-report questionnaire

Self-report questionnaire will be filled by participants directly on the website and their responses associate with their unique ID-code. Instruments used and timeline are reported in Table 1.

**Table 1.**
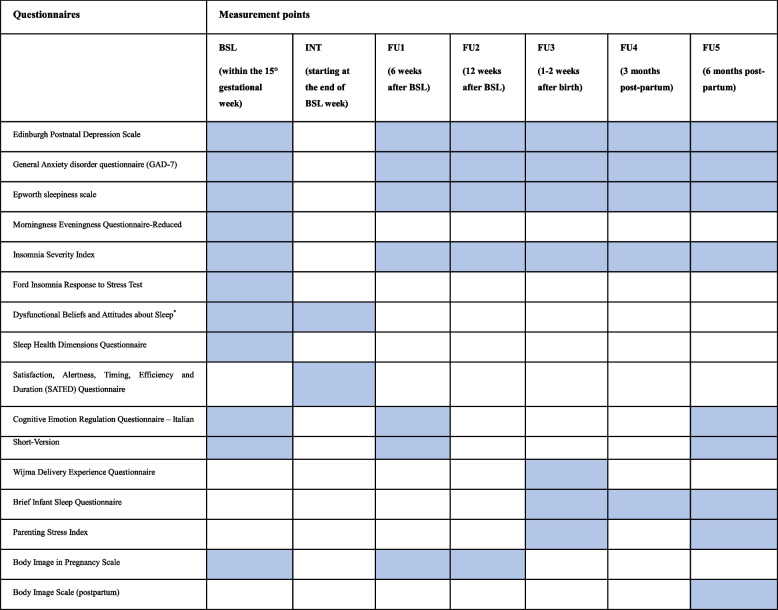
Questionnaire and measurement points

*Abbreviations*: *BSL *Baseline, *INT *Intervention, *FU F*ollow-up

*Subgroup B2 will complete the Dysfunctional Beliefs and Attitudes about sleep during session 3 of the intervention

#### EMA week

At Baseline, Follow-up 1 and Follow-up 5 participants will be observed for a 7-day EMA procedure.

**Fig. 3:**
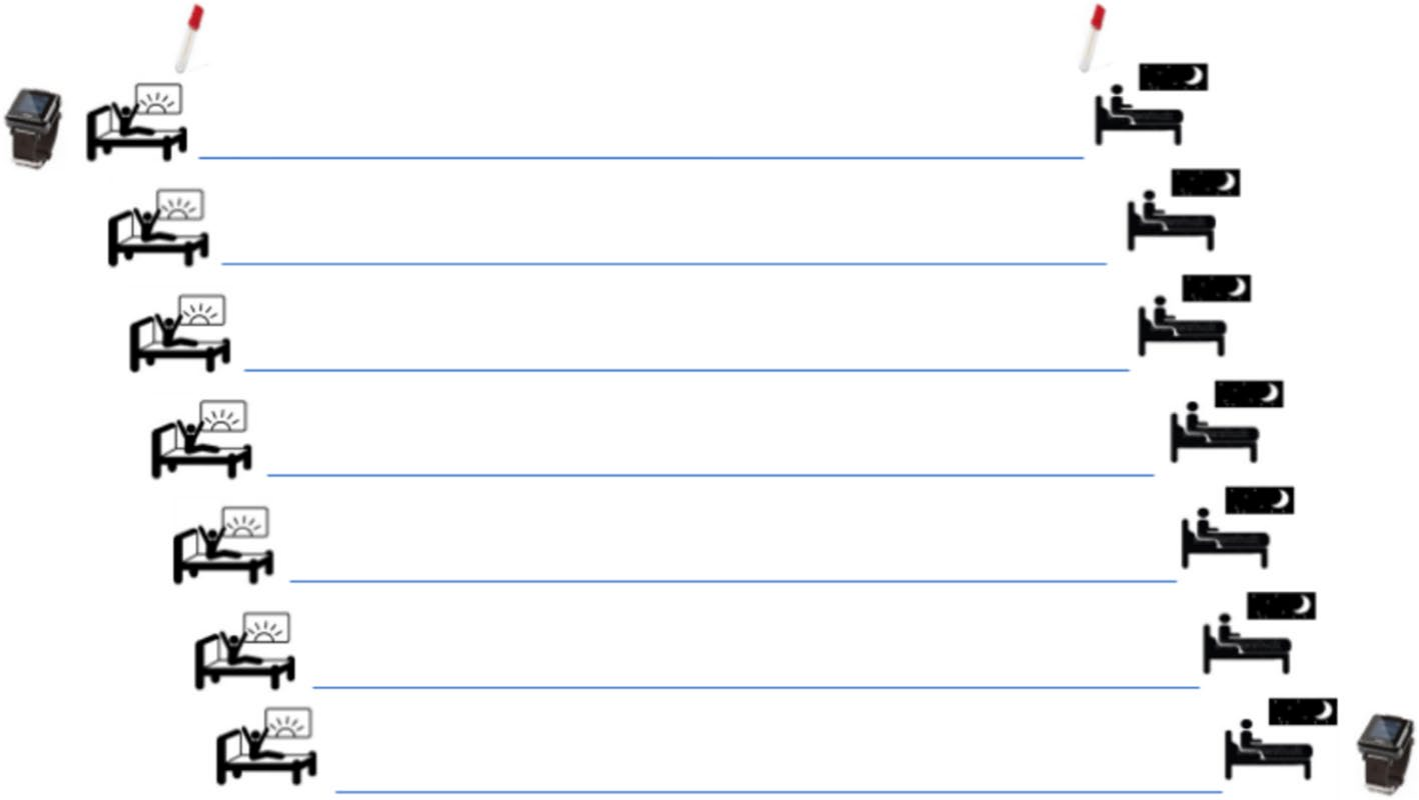
Graphic outline of the procedure for the ecological-momentary-assessment (EMA) week

The procedure is synthetized in Fig. 3 and include the following assessments:

#### Sleep and emotion diaries

The sleep diary will be filled online through the website and is developed in accordance with the norms for the “consensus sleep diary” [47]. The sleep diary is a calendar-style form in which information about sleep is reported every morning within 30 minutes of the last awakening and every evening at bedtime. The diary will include further assessment of emotional processes associated with sleep. Sleep variables will include: sleep onset latency (SOL), wake after sleep onset (WASO), total sleep time (TST), time in bed (TIB), sleep efficiency index (SEI). Moreover, the level of alertness (low energy-high energy) and the valence (positive-negative) and arousal (calm-agitated) of affective states in the evening and in the morning will be collected through visual scales [48].

#### Wrist actigraphy

Women will be asked to wear an actigraphy in their non-dominant hand for 7 days, during both day- and night-time. The actigraphy is a small, computerized wristwatch-like device used to detect sleep-wake activity. The actigraphy is an ecological and non-invasive device, with different models validated to evaluate sleep (SOL, WASO, TST, TIB, SEI) and wake (mean physical activity) parameters [49]. We will use the Micro Motionlogger actigraph (Ambulatory Monitoring, Ardsley, NY, USA), which has good agreement with polysomnography [50] and is widely used to detect sleep-wake activities in different populations [51, 52]. Data from actigraphic recordings will be scored by two trained investigators (D.M, E.B.) following decision by consensus.

#### Salivary samples

Salivary samples will be self-collected at home from consenting participants through a salivary swab the first day of each EMA week in 2 time-points (morning and evening). Samples will be used to evaluate salivary cortisol levels by liquid chromatography-tandem mass spectrometry (LC-MS/MS). Cortisol levels will be used as index of stress reactivity. Moreover, a cytobrush will be used to collect exfoliated cells from oral mucosa (1 assessment per person) and used for the analysis of DNA methylation of the genes FKBP5, BDNF (brain derived neurotrophic factor), and NR3C1 (glucocorticoid receptor), by bisulfite next-generation sequencing. These genes have been validated as intergenerational stress-related gene [22–24, 53, 54]. Here we will test the DNA methylation pattern of these genes as a potential biomarker of prenatal poor sleep, which is considered a stressing factor.

Since saliva and blood methylation patters are largely overlapping [55], we will use saliva samples to validate a new and easily applicable biomarker of prenatal stress.

The two biomarkers of parental stress (cortisol levels and methylation of the genes FKBP5, BDNF, and NR3C1) will be evaluated as outcome for the psychological intervention for insomnia.

### Compensation

All participants will receive compensation for their participations, which will be graduated based on phases’ completion for a maximum of 60 euros for each participant. Compensations will be divided as follows:

- drop-out after baseline: €5;
- drop-out after follow-up 1: €15;
- drop-out after follow-up 2: €30;
- drop-out after follow-up 3: €40;
- drop-out after follow-up 4: €50;
- completion of follow-up 5: €60.

This procedure is considered important to maximize motivations and to reduce the risk of drop-outs.

All participants will receive, at the end of the study, the following informative materials: the pdf materials of the fifth intervention session on infant’s and postpartum sleep; a pdf containing information on children sleep from 0 to 5 years.

### Ethical aspects

The trial was approved by the by Ethics Committee for psychological studies of the Guglielmo Marconi University of Rome, Italy (Approval letter: 19.02.2024) and the Bioethics Committee of the University of Bologna, Italy (ID: 0388063).

In case of important protocol modifications, amendments will be submitted for approval by the Ethics Committee for psychological studies of the Guglielmo Marconi University and the Bioethics Committee of the University of Bologna. The trial protocol registered with Clinicaltrials.gov will be updated accordingly.

Participants’ data will be maintained in secure spaces accessible only by the investigators. Signed informed consents and salivary samples will be stored in secure rooms at the study sites for the duration of the study. All study data will be entered in a database, labelled with the ID-codes. Only the study investigators will have access to the database.

### Statistical analysis

Analyses will be performed by a professional biostatistician (S.Cu.). For all outcomes, intent-to-treat analyses (ITT) will be performed including all randomized women in the statistical analyses. Descriptive statics will be reported, including recruitment rate, dropout rate, and data completion rate. A detailed flow chart of study participants will be provided. Only data from those completing the trail will be analysed. Baseline characteristics of non-completers and completers will be compared. Independent t-test and Chi-square test will be conducted to compare randomized groups on baseline characteristics. Significant differences between dropouts and retained participants will be investigated with comparisons on baseline and outcome characteristics. Multivariate linear regressions will be used for testing associations with insomnia during pregnancy and postpartum depression. A multivariate mixed effects model analysis will be performed to test the interventions efficacy over time. Within- and between-groups differences will be measured including as factors the Group (A, B1, B2) and TIME (Pre-; Post-; last weeks of pregnancy; Birth; 3; 6) and as outcome all endpoints which will be collected at all assessments. For all analyses statistical significance is set at p-values < 0.05.

### Dissemination plan

All study findings will be disseminated in anonymous and aggregated forms. Key findings of the study will be disseminated to academic (presentation at conferences, publication in high impact journals) and non-academic (websites of psychological and medical associations, social media) audiences. If the trial substantiates the efficacy of the psychoeducation intervention, medical services for pregnancy and peripartum care will be contacted to explore opportunities to implement and disseminate the intervention to pregnant women suffering from insomnia symptoms.

### Expected results and discussion

Women are at higher risk than men to develop psychopathological problems, including chronic sleep difficulties [2]. This gap is particularly relevant during specific women’s life period, i.e. puberty, peripartum, and menopause [56]. The peripartum represent a stressful period marked by societal, physiological, and hormonal changes, the adaptation to which can have long lasting consequences on the women, the newborn, and the whole family system [25]. The need to promote maternal mental health has been included in the World Health Organization’s agenda, and several papers have stressed the importance of sleep health for peripartum wellbeing [14, 15, 57]. Recently, there is a grows of clinical trials based on CBT-I for pregnant women with promising results [25]. Here we propose MOTHERS as a new preventive approach to promote sleep health in pregnant women, and to prevent the biopsychological cascade of events that can lead to negative pregnancy outcomes, post-partum maternal psychopathology, and children’s sleep impairments.

Three groups of women will be compared longitudinally: one group (B2) with sleep difficulties randomized to the psychoeducational intervention, one active control group (B1) with sleep difficulties randomized to an educational placebo intervention, and a group (A) without sleep difficulties serving as a passive control group. We expect that women in the intervention group will show, compared to the placebo group, post-intervention significant improvement in physiological (actigraphy) and self-report (questionnaire, sleep diaries) indices of sleep health as well as of stress reactivity (salivary cortisol levels) and psychopathology (self-report). MOTHERS includes modules directed to promote evidence-based self-management of sleep health, delivering basic knowledge about sleep during pregnancy, factors inhibiting or promoting sleep heath, and management of sleep in the postpartum as a family issue. The importance of sleep health as a positive and multidimensional concept is a key content delivered in the intervention, as each dimension of sleep health can have negative consequences on overall health [58]. While research has primarily focused on the effect of sleep duration on pregnancy outcomes [59], other dimensions are also important. For instance, sleep timing is commonly changed during pregnancy, but the effects of this changes are rarely considered. Fatigue is common during pregnancy, however sleepiness and fatigue are different constructs, and it is important to recognize this difference, as going to bed when still alert weaken the bed-sleep association. With knowledge of sleep health dimensions as a continuum, the person can be instructed to identify specific areas of vulnerability (e.g., late sleep timing) and strengths.

Programs aimed at improving sleep health are often focused on sleep hygiene [60, 61]. Sleep Hygiene Education (SHE) aims at providing information about factors interfering with sleep, including lifestyle factors (e.g., diet and substance use) and environmental factors (e.g., noise, temperature). While SHE is included in standard CBT-I protocols, it is not recommended as a standalone intervention for clinical sleep difficulties [26]. The use of SHE to promote sleep health in the general population is also met with some challenges and limitations. While some recommendations, such as care of the sleep environment and regular bedtime, are important to deliver in preventive programs, they may not be enough for people with already basic knowledge of sleep-interfering factors [26]. Moreover, SHE programs seldom include self-monitoring of sleep (e.g., through diaries), which is an important component of sleep improvement [62]. SHE also does not include a focus on perpetuating factors of insomnia disorders, i.e. cognitive, emotional, and behavioural factors, which can influence sleep-wake regulation [63–69].

MOTHERS delivers basic information on sleep regulation and psychological (cognitive, emotional, and behavioural) factors influencing sleep. The intervention provides evidence-based recommendation to manage sleep-interfering factors, including CBT-I derived strategies. MOTHERS also covers transversal competence, such as emotion regulation and management of everyday worries, to modulate cognitive and emotional factors which are associated with chronic insomnia disorder [70, 71] and a range of psychopathological conditions [72, 73]. The RCT protocol includes the use of sleep diaries during the 6 weeks of intervention, providing women the opportunity to self-monitor sleep-wake activities.

We expect that improvement in sleep health would lead to improvement in biological and self-report indices of wellbeing in the post-partum. Indeed, sleep difficulties in the peripartum increase the risk of perinatal depression (PND) and anxiety [15]. Moreover, sleep difficulties represent stressful experiences, which can alter the regulation of the stress response during pregnancy [21]. These alterations, in turn, can trigger epigenetic mechanisms and thus influence the stress regulatory systems of newborn with potential long-lasting effect on sleep regulation and psychopathology [21]. This first study will focus on women who reports no use of alcohol and smoking during pregnancy and no somatic or mental comorbidities, as these factors may influence and alter sleep. It should be noted that these same factors are associated with increased risk of PND [74]. If the present trial will have the expected results, MOTHERS could be tested in women with insomnia presenting risk factors for PND and thus more vulnerable to post-partum psychopathology.

Digital interventions are particularly suitable to pregnant women, as they break down barriers to psychological interventions, such as managing other prenatal health appointments and preserving personal time off for maternity leave [33]. There is thus a need for evidence-based psychoeducational programs which can be delivered digitally or in-person in the context of routine care of pregnant women. Sleep health is an important component, as it is routinely done with nutrition and physical activity. MOTHERS is intended to be delivered digitally but it is also adaptable as an in-person program. Moreover, women in the intervention conditions will also have the weekly opportunity, during the interventions, to have direct contact with an expert CBT-I clinician (C.B.) to express difficulties with the intervention’s contents and to have personalized feedback about their specific symptomatology. This is expected to have positive effects on women with specific difficulties and to increase adherence to the intervention, as adherence to guided digital CBT (dCBT) is higher than to unguided programs [75]. Moreover, when possible guided dCBT-I is preferable, as evidence suggest its superior efficacy to unguided dCBT-I [76, 77].

In conclusions, MOTHERS has several strengths and implications for familiar wellbeing in the peripartum. The intervention is innovative as it focuses not on a restricted series of sleep hygiene rules, which may be not appropriate for some individuals, but on overall promotion of sleep health, delivering knowledge and promoting attitude to self-manage sleep health. It is founded on the concept of sleep health as a positive construct [58] and on the 5 principles of good sleep health, which are universally adoptable and thus suited for different conditions [78]. It also includes basic information derived from models of insomnia disorder about behavioural, emotional, and cognitive processes influencing sleep health, and adapted CBT-I techniques to modify sleep-disrupting processes. The study includes assessments at the biological (salivary sample), physiological (actigraphy), and self-report data to broader the understanding of the multiple complex impact of sleep health and its promotion during pregnancy. We also included a pre- and post-intervention assessment and 4 follow-ups to tackle short- and long-term effects of the MOTHERS programs and the longitudinal psychobiological changes in sleep and wellbeing across pregnancy. The inclusion of assessment of partners’ and children’s sleep and wellbeing aims to further deepen the understating of sleep and psychopathology in the family context. Furthermore, intergenerational effects of maternal sleep and stress on children’s sleep problems and overall health will be assessed though questionnaire and diaries, and a new biomarker of maternal poor sleep as a stressing condition will be tested (the DNA methylation of the FKBP5, BNDF, and NR3C1 genes).

### Supplementary Information

Supplementary Material 1.

Supplementary Material 2.

## Data Availability

No datasets were generated or analysed during the current study.
