# Supplementary material for: Promoting sleep health during pregnancy for enhancing women’s health: a longitudinal randomized controlled trial combining biological, physiological and psychological measures, Maternal Outcome after THERapy for Sleep (MOTHERS)
Source: BMC Psychol. 2024 Jun 10;12:340. doi: 10.1186/s40359-024-01827-1 (PMC11165884; doi:10.1186/s40359-024-01827-1)
Supplement: Supplementary file 2 — Supplementary Material 2. [file 40359_2024_1827_MOESM2_ESM.pdf]

Supplemental File S2. Contents of intervention's sessions and associated book's chapters.

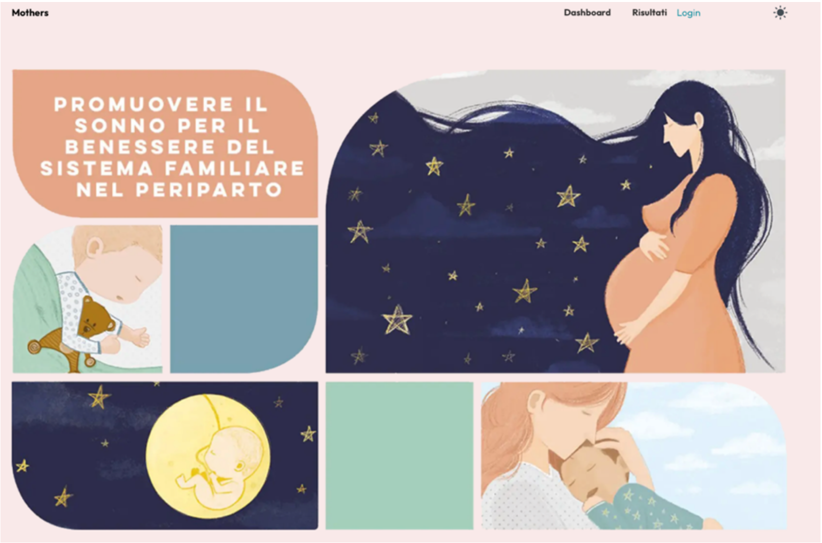

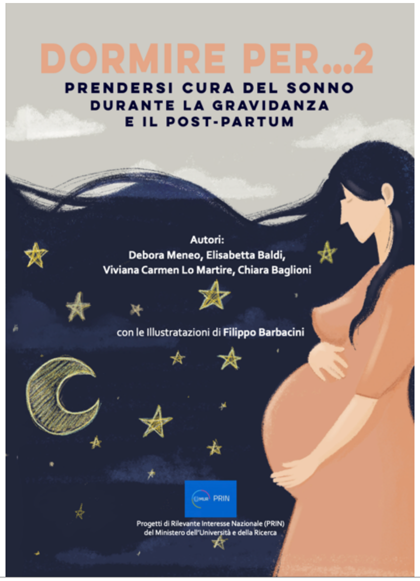

Intervention's sessions	Book's chapters
Session 1. Sleep during pregnancy: presenting the aims of the intervention and introducing psychoeducation on physiological regulation of sleep, on sleep health and on how sleep changes during pregnancy.	*Introduction*<br>*Chapter 1. How do you sleep during pregnancy?*
Session 2. Pre-sleep behaviours and habits: psychoeducation on psychological regulation of sleep and on the impact of behaviors on sleep regulation; introducing sleep compression and stimulus control.	*Chapter 2. How important for sleep are our pre-sleep behaviours and habits, particularly in pregnancy?*

Session 3. Beliefs about sleep: psychoeducation on cognitive factors maintaining sleep difficulties; introducing cognitive restructuring.	*Chapter 3. How true are our beliefs about sleep?*
Session 4. Sleep and emotions: psychoeducation on emotional factors maintaining sleep difficulties and on the bidirectional association between sleep and emotions; introducing emotion regulation techniques.	*Chapter 4: How much do emotions and sleep influence each other, particularly during pregnancy?*
Session 5. Children’s sleep: psychoeducation on sleep in the postpartum and on the development of sleep regulation in children; introducing bedtime routines.	*Chapter 5. How do babies sleep?*
Session 6. Value and personalize sleep: relapse prevention and focus on acquired skills and on how to prioritize sleep; introducing the 5 principles of good sleep health.	*Chapter 6. Valuing and personalising one's sleep to feel better: sleep and well-being of mother, child, and the whole family*
